# Targeting optimal PD management in children: what have we learned from the IPPN registry?

**DOI:** 10.1007/s00467-020-04598-0

**Published:** 2020-05-27

**Authors:** Dagmara Borzych-Dużałka, Franz Schaefer, Bradley A. Warady

**Affiliations:** 1 Department of Pediatrics, Nephrology and Hypertension, Medical University of Gdańsk, Gdańsk, Poland; 2 Center for Pediatrics and Adolescent Medicine, Heidelberg, Germany; 3 Children’s Mercy Hospital, Kansas City, KS USA

**Keywords:** Pediatric end-stage kidney disease, Renal replacement therapy, Peritoneal dialysis, International registry

## Abstract

National and international registries have great potential for providing data that describe disease burden, treatments, and outcomes especially in rare diseases. In the setting of pediatric end-stage renal disease (ESRD), the available data are limited to highly developed countries, whereas the lack of data from emerging economies blurs the global perspective. In order to improve the pediatric dialysis care worldwide, provide global benchmarking of pediatric dialysis outcome, and assign useful tools and management algorithms based on evidence-based medicine, the International Pediatric Peritoneal Dialysis Network (IPPN) was established in 2007. In recent years, the Registry has provided comprehensive data on relevant clinical issues in pediatric peritoneal dialysis patients including nutritional status, growth, cardiovascular disease, anemia management, mineral and bone disorders, preservation of residual kidney function, access-related complications, and impact of associated comorbidities. A unique feature of the registry is the ability to compare practices and outcomes between countries and world regions. In the current review, we describe study design and collection methods, summarize the core IPPN findings based on its 12-year experience and 13 publications, and discuss the future perspective.

## Introduction

End-stage kidney disease (ESKD) is a rare condition in childhood with an annual incidence ranging from 4 to 14 and a prevalence from 18 to 100 per million age-related population [1, 2]. The reason for this variation is multifactorial and can be explained both by non-modifiable ethnic and genetic factors, as well as by differences in national wealth and healthcare organization expenditures, which affect access to renal replacement therapies (RRT) including kidney transplantation, peritoneal dialysis, and hemodialysis. Nevertheless, according to the RRT registry of the International Pediatric Nephrology Association (IPNA), chronic RRT is available to children in 84 countries, which covers 81% of the world’s childhood population [3]. As RRT is a lifelong treatment, strict attention should be given to strategies designed to optimize this care with a goal to prolong each and every patient’s lifespan and quality of life. Although transplantation is the treatment of choice for all children with ESKD, it is not always feasible immediately and dialysis is typically the initial RRT modality prescribed. Due to its unique compatibility with the lifestyle of most families, peritoneal dialysis (PD) remains the initial treatment of choice for most pediatric ESKD patients, especially in the pre-adolescent population [4].

In spite of the fact that the majority (62.5%) of children undergoing chronic PD live outside Europe and North America, most of the available epidemiological data on pediatric PD has been derived from the United States Renal Data System (USRDS), The North American Pediatric Renal Trials and Collaborative Studies (NAPRTCS), and the European ESPN/ERA-EDTA registry, providing information limited to highly developed countries. In contrast, there is limited information about dialysis performance in many developing countries, a reality that has blurred the global perspective [3–6].

To address the need for a more global perspective on PD and to facilitate collaborative international research in pediatric dialysis care, the International Pediatric Peritoneal Dialysis Network (IPPN) was established in 2007. The aims of this initiative were to (i) collect comprehensive information about pediatric dialysis care worldwide, (ii) provide useful tools and management algorithms for daily clinical practice, (iii) provide global benchmarking of pediatric dialysis outcomes, and (iv) provide a basis to perform prospective observational studies in pediatric dialysis. In 2012, the initiative was renamed the “International Pediatric Dialysis Network (IPDN)” following the addition of a separate registry for children undergoing chronic hemodialysis (the International Pediatric Hemodialysis Network (IPHN) Registry). To date, 127 centers from 43 countries actively contribute patient-related data, resulting in the largest pediatric dialysis dataset on a global level (Table 1). Since 2007, over 3700 PD patients and 900 HD patients have been enrolled.

**Table 1:** Centers and patients enrolled in IPPN registry (as of April 2020)

Region/country	Participating centers	Cumulative pt number
Europe	48	1260
Austria	1	6
Belgium	1	25
Czech Republic	1	69
Germany	10	281
Finland	1	51
France	8	139
Great Britain	2	190
Greece	2	44
Hungary	1	41
Italy	5	119
Lithuania	1	22
Macedonia	1	16
The Netherlands	2	10
Poland	5	186
Romania	1	9
Spain	4	40
Sweden	2	12
Turkey	8	319
Asia/Middle East	22	1087
China	2	346
Hong Kong	1	47
India	7	113
Iran	1	82
Israel	1	7
Lebanon	1	4
Malaysia	1	70
Oman	1	18
The Philippines	1	88
Saudi Arabia	1	49
Singapore	1	58
South Korea	2	157
United Arab Emirates	2	48
North America	24	458
Canada	2	74
USA	22	385
Latin America	23	701
Argentina	6	180
Brazil	2	19
Chile	8	216
Colombia	2	40
Mexico	1	27
Nicaragua	1	28
Peru	2	128
Uruguay	1	61
Oceania	1	68
New Zealand	1	68

## Registry design and data collection

The IPPN registry collects prospective patient-related information exclusively via an Internet-based web platform (www.pedpd.org). The participating centers are asked to enroll all incident and prevalent PD patients and to enter data longitudinally until chronic PD is discontinued.

The database comprises basic patient-related demographics including birth weight and height, primary renal disorder, associated comorbid and genetic conditions, data on previous renal replacement treatment (RRT), age at PD initiation, catheter type and placement technique, presence of any ostomies, and patient/caregiver Staphylococus aureus carrier status. Six-month updates include information about any hospitalizations within the observation period, clinical status including anthropometry, Tanner pubertal status, blood pressure, urine output, PD ultrafiltration, nutritional supplementation, signs and symptoms of bone disease, tissue calcification and uremia, dialysis modality scheme, PD catheter exit site scoring, detailed biochemistry parameters, and medications. Optional cardiovascular assessment includes echocardiographic parameters and 24-h blood pressure data. In addition, peritoneal equilibration test and clearance data are collected, and detailed peritonitis, exit site infection, and access revision information are entered whenever such an event occurs. Termination data is requested whenever a patient discontinues PD which provides detailed information about the reason for PD cessation including HD transfer, type of transplantation (living related or deceased donor), or death.

All data entries are automatically checked for plausibility and completeness. In addition, a manual check is performed each time at database upload. When doubts arise pertaining to data quality, accurateness, or consistency, the center’s lead investigator is contacted and the submitted data are discussed per e-mail or by phone.

Data protection is ensured by pseudonymized data entry. The registry protocol is approved by the ethical committee/institutional review board as required at each participating center. Written parental consent and, whenever appropriate, assent from patients is obtained from all participants.

## Coordinators, investigators, and funding of the registry

The IPPN registry is coordinated by two Principal Investigators (B.W. and F.S.) and a Coordinating Physician (D.B.D.). Statistical analyses are performed by a team of biostatisticians.

Data entry is performed by participating center physicians, nurses, or data entry personnel. Each center has a lead investigator and co-investigators. Every IPPN member has a unique username and password for data entry.

The project is funded by commercial sponsors and the International Society for Peritoneal Dialysis (ISPD).

## Clinical lessons learned from the IPPN

### Economic perspective

Economic welfare is the key determinant of health and access to healthcare services. Limited access to diagnostic tools and inadequate surveillance systems in low-income countries have been shown to influence CKD detection, monitoring, and management of adult ESKD populations [7]. This same scenario impacts pediatric patients. Whereas in Europe, North America, and Japan, RRT is offered to virtually all children with ESKD whenever required, there are large parts of the world, including most African and many Asian regions, where due to the paucity of centers providing RRT and high treatment costs, dialysis is accessible only by the wealthiest members of society. For instance, in India and China, the estimated prevalence of pediatric RRT is less than 10% of that observed in Western countries, likely evidence that only a small fraction of children requiring RRT have access to it [3]. Similarly, a recent review assessing access to RRT in sub-Saharan Africa revealed that only 61% of children who required chronic dialysis received at least one session, while only 35% remained on dialysis for more than 3 months. This was the result of the inability of parents to pay for expensive treatment, as the costs of chronic dialysis are not covered by the public health service in this region [8]. An analysis of IPPN data from 2012 provided a unique opportunity to identify the impact of economic conditions on PD practices and outcomes at a global level [9]. Over 1700 PD patients from 33 countries were classified based on the gross national income (GNI) as coming from high (47%), upper-middle (11%), lower middle (34%), and low-income (8%) countries. Data analysis revealed a close positive association between GNI and the number of infants and children with comorbidities who received dialysis, suggesting that access to RRT therapies is indeed limited by a country’s economic resources and that in a setting of severely limited resources, access to dialysis tends to be restricted to pediatric patients with isolated kidney failure and a lower risk of complications related to comorbidities or PD-related technical difficulties. Similarly, the primary renal disorder was reported as unknown in almost one fourth of patients from low-income countries, arguing that the lack of resources and inability to perform extensive diagnostic testing likely resulted in late referral practices in those settings where dialysis could be performed and an associated increased risk for clinical comorbidities in many instances. The GNI also affected the prevalence of enteral tube feeding and treatment with calcium-free phosphate binders, active vitamin D, and erythropoietin-stimulating agents (ESA), which not unexpectedly influenced treatment outcomes. As a result, patients from lower income countries had significantly higher serum parathyroid hormone levels and lower serum calcium and hemoglobin concentrations. Use of recombinant growth hormone (rhGH) therapy was largely restricted to high- and middle-income countries and the GNI explained almost 40% of the variation in height. The use of pH-neutral PD solutions increased progressively with GNI from 8% in the low-income countries to 48% in the high-income countries. Excluding patients from the United States, where such fluids are not approved by the FDA, the use of biocompatible PD fluids was even greater, at 68% of patients in the high-income economies.

**Fig. 1:**
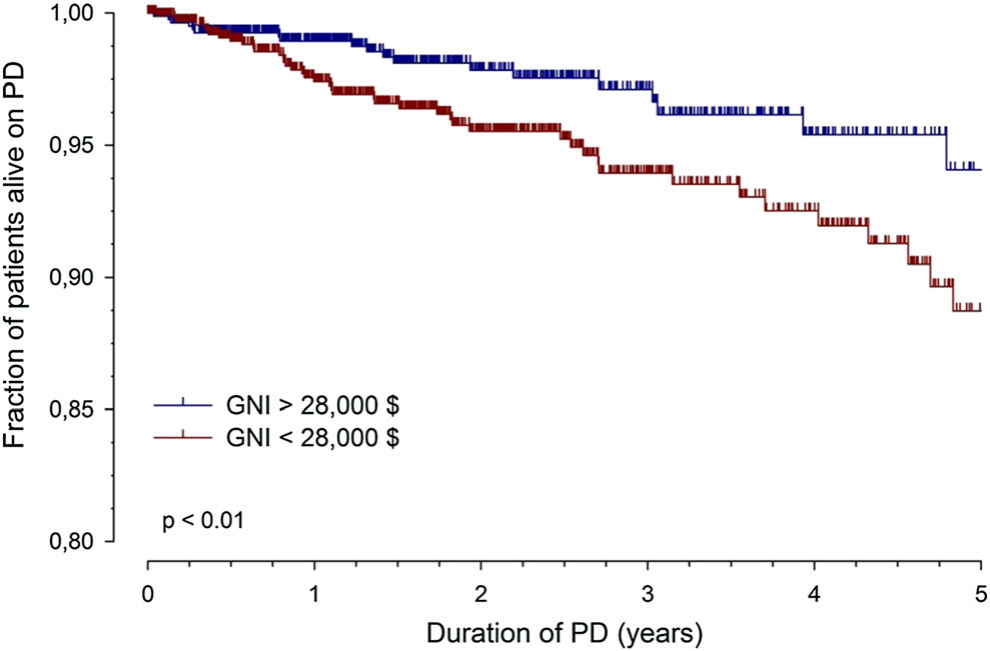
Patient survival in countries with CNI per capita greater or less than 28,000 $ per year (figure adapted from 9; used with permission)

Most importantly, patient mortality was strongly affected by GNI (HR per $10.000: 3.3, CI 2.0 to 5.5), independent of patient age and the presence of comorbidities (Fig. 1). The most recent IPPN study focusing on mortality in children treated with PD confirmed the previous findings and showed substantial regional variation in patient survival, with GNI explaining 50% of the mortality risk. The 3-year probability of death ranged from 2% in North America to 9% in Eastern Europe. Other major explanatory factors included CAPD modality at dialysis initiation, low BMI, young age, female sex, primary renal disorder other than CAKUT, and the presence of comorbidities [10].

### Nutritional status and growth

**Fig. 2:**
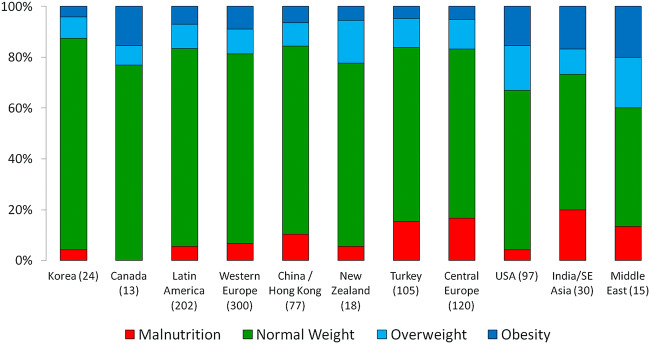
Regional variation of nutritional status at start of CPD, sorted by decreasing fraction of patients with BMI within normal range (figure adapted from 13; used with permission)

**Fig. 3:**
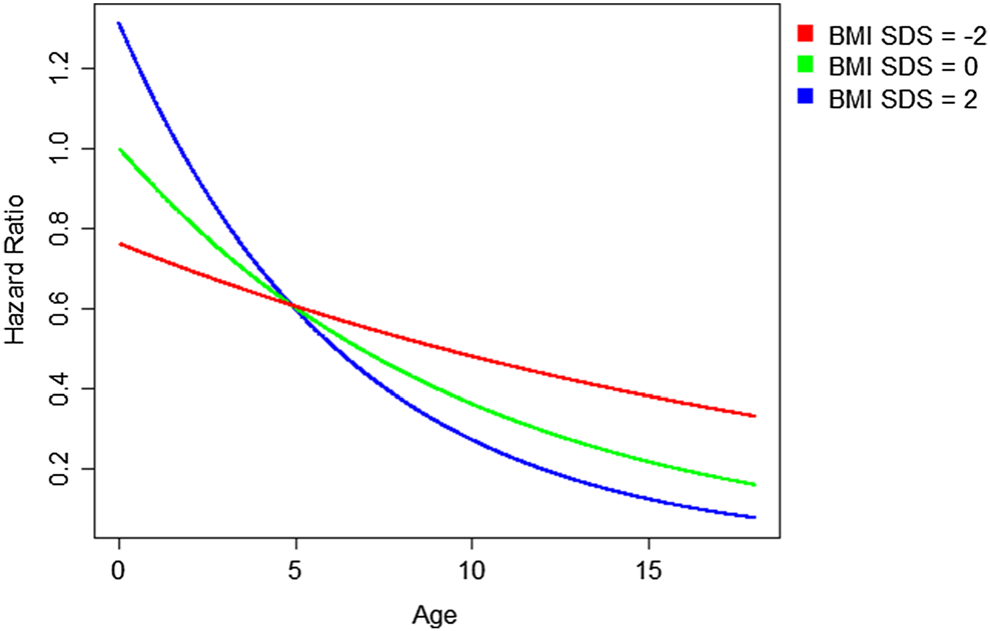
Hazard ratios of death according to age and BMI SDS (reference: age = 0, BMI SDS = 0) for a patient with no comorbidities, based on Cox regression with time-dependent variables age, BMI SDS, presence of comorbidities and the interaction of BMI SDS and age (figure adapted from 13; used with permission)

The majority of published studies assessing the nutritional status of dialyzed children have been performed at highly specialized pediatric dialysis programs in North America and Western Europe. These data have resulted in widely referenced guidelines [11, 12]. However, the risk of nutritional abnormalities in individual regions and countries on a global scale is affected by a range of unique medical and non-medical factors including patient ethnicity, underlying disease and comorbidities, national healthcare expenditures, cultural acceptability of dietary and feeding prescriptions, availability of special formulas, and enteral feeding equipment, all in addition to differences in local, national, or regional nutritional recommendations [12]. In an analysis of the nutritional status of 1001 PD patients enrolled in the IPPN registry, the overall prevalence of underweight and overweight/obesity at the start of CPD was 8.9% and 19.7%, respectively, confirming that the worldwide trend towards overweight/obesity also includes the pediatric ESKD population [13]. Malnutrition was found to be most common in South and Southeast Asia (20%), Central Europe (16.7%), and Turkey (15.2%), whereas overweight and obese patients were most prevalent in the Middle East (40%) and the United States (33%) (Fig. 2). With recognition that obesity in the general population has been linked to the development of hypertension, insulin resistance, impaired glucose tolerance, high cholesterol, and increased overall mortality, the potential adverse outcome for patients with the additional burden of ESKD is of great concern. In addition, an interesting interaction between BMI and age with regards to survival was demonstrated. While in infancy, mortality risk was preferentially amplified by obesity, in older children, mortality was markedly increased in those with malnutrition (Fig. 3).

Poor growth and failure to thrive remain a substantial treatment challenge unique to pediatric dialysis care. Not only does short stature have a negative impact on quality of life both during childhood and adulthood, but it has also been associated with patient mortality [14, 15]. The etiology of growth failure in CKD is multifactorial and correlates with the degree of kidney dysfunction [16]. Major contributing factors include the accumulation of inhibitors of growth hormone (GH) and insulin-like growth factor (IGF)-1 signaling, the malnutrition-inflammation complex, metabolic acidosis, anemia, salt depletion, and hyperparathyroidism [17]. It remains somewhat frustrating that poor growth persists despite the recognition that growth can be efficiently promoted in all stages of CKD by the provision of adequate nutrition, correction of electrolyte and acid-base abnormalities, aggressive treatment of CKD-MBD and anemia, and recombinant growth hormone use. In the IPPN cohort, the mean height standard deviation score (HtSDS) has been found to be below − 2 SDS and varies worldwide between − 1.4 and − 3.5 SDS. Despite the high prevalence of short stature, the overall rhGH usage has been only 15% in growth-retarded pre-pubertal children who are candidates for the therapy [18]. This is partially due to its high cost, accompanied by legal restrictions pertaining to its prescription in some countries [19].

As one third of postnatal growth occurs within the first 2 years of life, an analysis of the growth and nutrition of 153 infants < 24 months of age enrolled in the IPPN registry was conducted [20]. The combination of comprehensive prospective data collection, coupled with a milestone analysis of length and weight change during the course of dialysis, provided a complete picture of the early infantile growth pattern associated with the provision of PD. The need for supplementary feeding in young infants to achieve optimal weight gain was clearly demonstrated. However, the core finding of this analysis was the superiority of gastrostomy (GS) over nasogastric (NG) tube feeding in terms of improving infantile growth, with the fraction of observation time spent with GS feeding being an independent predictor of longitudinal growth. Whereas both feeding methods positively impacted nutritional status, only GS feeding was associated with catch-up growth. The most likely explanation for this finding is a reduction in vomiting in gastrostomy-fed children. Of note, gastrostomy feeding was almost exclusively used in the US and Europe. In addition, the use of biocompatible PD solutions and the early initiation of rhGH therapy were also associated with improved linear growth [20].

### Anemia management

**Fig. 4:**
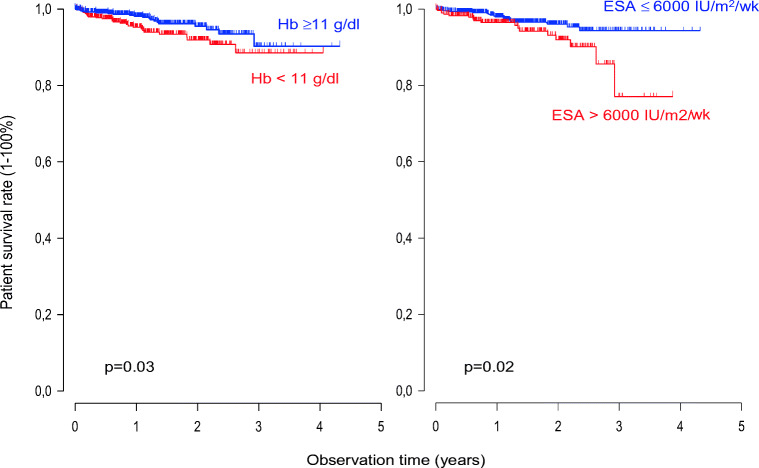
Kaplan-Meier actuarial survival curves for patients with mean Hb greater or less than 11 g/dl (left panel), and those with mean administered ESA equivalent dose greater or less than 6000 IU/m^2^ per week (figure adapted from 27; used with permission)

Anemia in patients with CKD/ESKD results from the impaired production of erythropoietin, iron deficiency, blood loss, and reduced erythrocyte lifespan. Additional contributing factors include a persistent pro-inflammatory state, hypervolemia and hyperparathyroidism. Anemia leads to fatigue, depression, sleep disturbance, impaired cognition, loss of appetite, and decreased exercise tolerance [21]. Optimal anemia management in the pediatric ESKD patient is particularly important because of their desire for regular physical activity, the need for optimal cognitive functioning at school, and the mandate to limit exposure to blood products so as to increase the likelihood of transplantation [22]. Treatment of CKD-associated anemia primarily consists of erythropoiesis-stimulating agents (ESAs) and iron [23]. In the context of findings streaming from large prospective trials in adults linking the normalization of hemoglobin and high ESA dosing with adverse outcomes and increased mortality [24–26], the IPPN database was interrogated to gain insight into anemia treatment paradigms in dialyzed children. In 2013, data from 1394 children on PD from 30 countries was reviewed [27]. It was noted that despite the finding that ESAs were prescribed to more than 90% of patients, one fourth of children had hemoglobin (HB) levels below target. As might be expected in an analysis of global practice, significant regional variation was observed, with European and North American centers generally achieving higher HB values than centers in Asia, Turkey, and some Latin American countries. The presence of anemia was associated with hyperparathyroidism*,* low urine output, hypoalbuminemia, hypertension, and left ventricular hypertrophy (LVH), the latter four clinical factors associated with hypervolemia in these PD patients. In addition, the ESA dose correlated inversely with the HB level, suggesting that ESA resistance, in part related to fluid overload rather than underdosing, is partially responsible for inefficient anemia management. Also noteworthy was the finding that ferritin levels were inversely correlated with HB levels, indicating that high serum ferritin reflects inflammation rather than appropriate iron storage. To address the all-important clinical question of the optimal HB concentration in children on PD, a comparison of different achieved HB ranges revealed a significant increase in patient mortality associated with a mean achieved HB < 11 g/dl, which is in line with published pediatric hemodialysis data, but in contrast to adult interventional trials in which high HB levels predicted cardiovascular events and overall mortality [24–26, 28]. The mechanisms underlying the adverse outcomes associated with full anemia correction in adults may include an increased risk of thrombus formation by hemoconcentration, as well as off-target effects of the high ESA doses including activation of endothelial cells and stimulated production of endothelin and plasminogen activator inhibitor-1 [29]. We also demonstrated for the first time in a pediatric population a positive association between ESA dosing and mortality [27]. When the ESA dose was normalized to body surface area in contrast to weight, an average weekly dose of 6000 IU/m^2^/week and higher was associated with an increased risk for mortality [27]. The link between high ESA dose and poor patient survival emphasizes the need for exploring and treating the causes of ESA resistance (i.e., chronic inflammation) in anemic pediatric PD patients. Figure 4 represents survival curves for patients with a mean Hb greater or less than 11 g/dl (left panel) and those with a mean administered ESA equivalent dose greater or less than 6000 IU/m^2^ per week.

### Cardiovascular disease

Cardiovascular disease remains the most frequent single cause of death in children on dialysis, accounting for 30% of all deaths [30]. The strongest predictors include the presence of hypertension, persistent fluid overload, altered mineral metabolism, and inflammation, which together lead to left ventricular hypertrophy (LVH), an independent risk factor and an intermediate end point of cardiovascular morbidity in patients with CKD/ESKD [31–33]. When assessing left ventricular mass (LVM), age, gender, body size, and composition must be considered because metabolic demand and cardiac perfusion vary with stature and gender, determining the physiologic adaptation of heart size. In turn, the reported prevalence of LVH in children with CKD/ESKD ranges from 17 to 80%, the variation being associated not only with CKD stage, but also with the definition of LVH used [33–35]. In an IPPN database analysis to address this issue, Borzych et al. reassessed and compared the effect of different methods to characterize LVH on the calculated prevalence of LVH [36]. The definitions used included (1) LVMI exceeding 38.6 g/m^2.7^ [37], (2) LVMI exceeding the 95th percentile for gender and chronological age according to Khoury et al. [38], (3) substituting chronological age by height age, and (4) LVM exceeding the 95th percentile (i.e., LVM SDS > 1.645) according to the reference charts provided by Foster et al. [39]. The analysis revealed that the prevalence of LVH did, in fact, range between 27.4 and 51.7%, depending on the scaling method used [36], suggesting the need for further studies to develop optimal normalization techniques to express LVM and other morphologic and functional measures across the pediatric age range.

In a subsequent IPPN study assessing risk factors for LVH, the most important determinants were blood pressure and volume status [40]. Bakkaloglu et al. demonstrated that the risk of LVH was more than doubled in children with systolic hypertension. Upon follow-up, systolic office BP was 7 mm higher in children who developed or had persistent LVH compared to children whose LVH regressed or who maintained normal LV morphology. The fact that some patients exhibited regression of LVH suggests that there may be some plasticity of the myocardium permitting improved geometry with more effective clinical management. Along the same lines, high total fluid output (sum of urine and PD-related ultrafiltration) was found to be protective from concentric geometry and patients undergoing automated PD, which has a higher fluid removal capacity than CAPD, appeared to be partially protected. In addition, the use of renin-angiotensin system (RAS) antagonists, independent of BP control and most likely by antagonizing the effect of local angiotensin II formation and the resultant prevention of myocyte hypertrophy, was also protective of concentric geometry. Among non-hemodynamic factors, high body mass index, presence of hyperparathyroidism, and an underlying renal disorder other than hypo/dysplasia were identified as independent predictors of LVH [40].

### Mineral and bone disorder

**Fig. 5:**
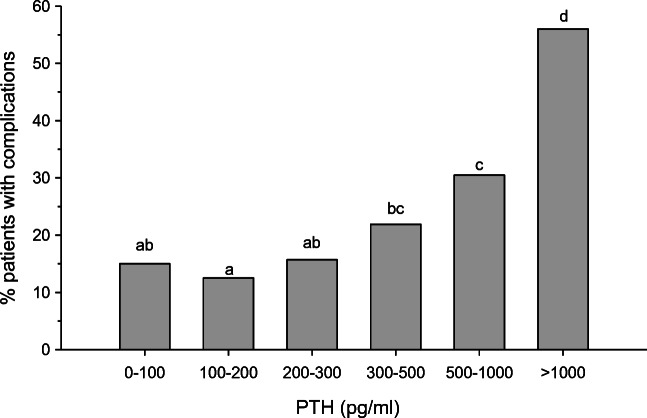
Percentage of patients with alterations of bone and mineral metabolism (bone pain, limb deformities, extraosseous calcifications, radiological osteomalacia, and/or osteopenia) stratified by time-averaged mean PTH levels. Groups sharing same letters do not differ significantly (figure adapted from 43; used with permission)

**Fig. 6:**
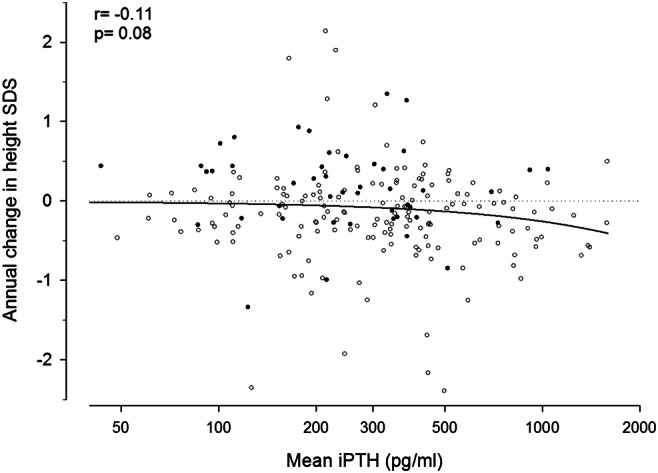
Time-averaged mean plasma iPTH concentrations and change in standardized height in 214 pre-pubertal children followed prospectively for at least 12 months. Full dots represent children treated with growth hormone (figure adapted from 43; used with permission)

Chronic kidney disease-mineral bone disorder (CKD-MBD) is a clinical issue of special concern in pediatric dialysis patients as alterations in calcium-phosphate metabolism and secondary hyperparathyroidism are associated with poor growth velocity and increased cardiovascular morbidity and mortality [41–43]. The aim of treatment is to promote normal bone turnover and mineralization and, in turn, to prevent skeletal deformities (including rickets), osteopenia, bone and joint pain, slipped epiphyses, muscle weakness, and fractures. In an IPPN survey on this topic, clinical symptoms and/or radiological signs of bone disease were observed in 15% of patients at registry entry and correlated with PTH levels (Fig. 5) [44]. In addition, about 22% of patients presented with an elevated serum calcium*phosphate product. Whereas bone biopsy findings were not available, evidence of adynamic bone disease according to surrogate indicators (i.e., elevated serum calcium, low PTH, and/or osteopenia) was present in 4.5% of patients. The prevalence of hyperphosphatemia markedly increased with age to more than 80% in adolescents; in contrast, hypophosphatemia was most common during infancy. PTH levels were outside the K/DOQI targets in 84% of patients, with almost even fractions lying above and below the target range. The prevalence, persistence, and de novo development of clinical and/or radiological signs of CKD-MBD were clearly associated with serum PTH levels more than 5 times ULN (i.e., > 300 pg/ml). The annual change in standardized height tended to be inversely correlated with the time integrated mean PTH level, and patients with a mean PTH > 500 pg/ml (i.e. > 9 times ULN) experienced a significant loss in height SDS as compared to children with lower PTH levels (Fig. 6). This analysis also provided evidence that PTH levels exceeding 200 pg/ml independently predicted LVH risk and values more than 500 pg/ml were associated with sub-target hemoglobin levels [44]. All these findings highlight the need for re-evaluation of CKD-MBD guidelines for children, including determination of the long sought after optimal PTH target in this population.

### Comorbidities

A superior survival rate reported in the pediatric, as compared to the adult ESKD population, is often attributed to a lower prevalence of comorbidities such as diabetes or cardiovascular disease (CVD) in this population. Nevertheless, pediatric patients do suffer from associated disorders, but of a different nature. Comorbid conditions commonly include genetic, familial, or congenital abnormalities. One third of the IPPN population has been documented to have had at least one comorbidity and its presence was found to be associated with a higher hospitalization rate and decreased patient survival [45]. The most common co-morbidity was cognitive impairment, present in 15.5% of patients. A cardiac or pulmonary abnormality was present in 9% and 4% of patients, respectively. Of the 150 patients with an identified syndrome (8.2%), a large proportion had at least 1 nonrenal comorbidity [45]. Although some of the comorbidities may be related to oligohydramnios or the early development of CKD in patients with CAKUT, the co-existence of these may also support the notion that genetic factors might play a role in both the primary kidney disorder and other organ malformations [46, 47]. A unique comorbidity investigated by the IPPN, and whose analysis benefited from the collaboration of the participating centers because of its rare nature in patients on PD, is meningomyelocele and use of a ventriculo-peritoneal shunt (VPS). The presence of such a foreign body in the peritoneal cavity is often believed to be a contraindication for safe and effective PD. In 18 PD patients with a concurrent VPS and peritoneal dialysis catheter identified by the IPPN, the peritonitis rate was 1/19.6 months, which is comparable to the rate of 1/18.8 months in the 2011 annual report of the North American Pediatric Renal Trials and Collaborative Studies (NAPRTCS) [5] and 1/21.6 months from unpublished IPPN data (Franz Schaefer–personal communication). In addition, there were no episodes of an ascending shunt infection or meningitis reported, suggesting that a VPS should not preclude PD as an option for children requiring renal replacement therapy [48].

Most recently, the IPPN also identified and reported on 20 children on CPD with a concurrent colostomy, which has historically been a very unusual scenario, but which is becoming more common in centers with aggressive neonatal management. Although CPD is feasible in these children, it has been associated with an increased risk of peritonitis. In the studied patient cohort, the annualized peritonitis rate was indeed higher in the colostomy patients as compared to a matched PD control group (1.13 vs 0.7). The experience has suggested that alternative strategies, such as placement of the PD catheter exit-site on the chest wall, may be necessary to decrease the risk of infectious complications in these complex patients [49].

### Residual kidney function

**Fig. 7:**
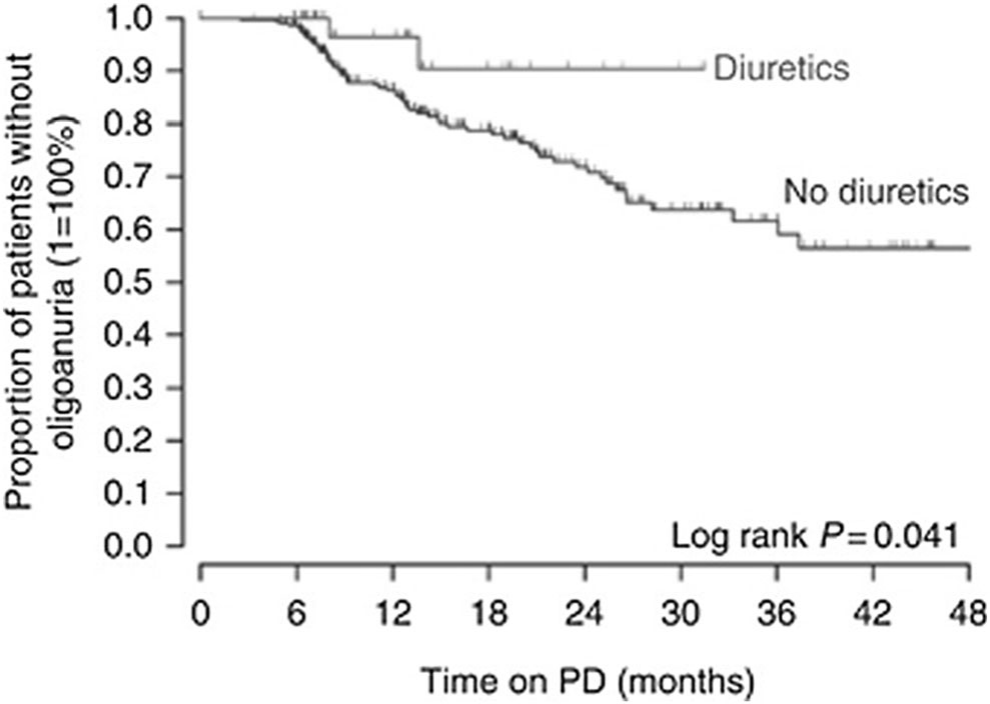
Survival of residual diuresis in patients receiving diuretic therapy compared with patients without diuretics (figure adapted from 50; used with permission)

In dialyzed adult patients, preservation of residual urine output has been associated with better survival, lower morbidity, and a greater quality of life. In the CANUSA study, each additional 250 ml of daily urine volume was associated with a reduction in the relative risk of death by 36% [50]. Ha et al. prospectively monitored urine output in 401 pediatric patients in the IPPN registry who commenced PD with significant daily residual diuresis (≥ 100 ml/m^2^/BSA) [51]. Daily urine volume subsided significantly more rapidly in children with glomerular disease, lower urine output at the start of PD, high ultrafiltration volume, and icodextrin use. Importantly, the administration of diuretics significantly reduced the oligoanuria risk (Fig. 7), whereas the prescription of renin–angiotensin system antagonists significantly increased the risk of oligoanuria.

### Peritoneal access revision

Successful PD requires a well-functioning peritoneal dialysis catheter (PDC). Among features potentially influencing catheter performance are catheter type, exit site orientation, placement technique, timing of first catheter use, and exit site care. Ideally, the placement technique, the characteristics of the PD catheter, and the care of the catheter should result in few mechanical or infectious complications.

Borzych-Dużałka et al. analyzed the risk factors for peritoneal access revision in the IPPN cohort [52]. In total, 452 access revisions were recorded in 321 (13%) of 2453 patients over 3134 patient-years of follow-up, resulting in an overall access revision rate of 0.14 per treatment year. Eighty-three percent of access revisions were reported within the first year of PD treatment. The main reasons for access revision included mechanical malfunction (60%), which predominated during the initial year of PD, followed by peritonitis (16%). The risk of access revision was increased in association with younger age, diagnosis of CAKUT, coexisting ostomies, presence of swan neck tunnel with curled intraperitoneal portion and, surprisingly, countries with higher gross national incomes. We speculate that the very unexpected discovery of a lower access revision risk in low-income countries might be related to closer adherence to international guidelines in recently established pediatric PD centers in developing countries. A key finding of the analysis was that the need for access revision increased the risk of PD technique failure or death (HR, 1.35; 95% CI, 1.10 to 1.65; *P* = 0.003). In addition, access dysfunction due to mechanical causes doubled the risk of technique failure compared with infectious causes (HR, 1.95; 95% CI, 1.20 to 2.30; *P* = 0.03). Whether the performance of PD placement by a limited number of surgeons at each site would result in fewer mechanical complications requires future study.

### Neonatal dialysis

In 2014, along with ESPN/ERA-EDTA Registry, The Australian and New Zealand Dialysis and Transplantation Registry and The Japanese Society for Dialysis Therapy Registry, the IPPN provided information on the survival and outcome of 264 patients who initiated chronic dialysis during the neonatal period [53]. Among these patients, the survival rate was 81% and 76% at 2 and 5 years, respectively. Neurologic disease was the most significant risk factor for mortality. These data provided evidence that chronic PD initiated in the neonatal period can be successful, despite major treatment challenges associated with comorbidities, infections, nutritional problems, hypertension, and growth failure.

### Future perspective

The need for collaborative international and inter-continental registries is constantly growing, especially in rare diseases. The recently established IPNA Global Renal Replacement Therapy (RRT) Registry has been developed to increase the quantity and the quality of demographic information on pediatric ESKD and RRT around the world [3]. In this endeavor, IPNA is partnering with regional, national, and international dialysis and transplant registries, and with individual pediatric dialysis centers wherever national registries are not available. Data collected by the IPDN, as well as by the registry of the European Society of Pediatric Nephrology will contribute to the IPNA registry. In addition, we are currently in process of finalizing data transfer agreements between IPNA, IPDN, and the NAPRTCS Registry. Once established, this will result in the largest and most comprehensive database of pediatric RRT worldwide.

## Limitations of the IPPN registry

Global online data collection has both strengths and limitations. Although the studies presented here only became possible as a result of the contribution of multiple pediatric dialysis centers around the globe, the voluntary nature of the registry and potential regional differences in PD populations and treatment practices might influence observed outcomes. We cannot entirely exclude selection bias related to the type of centers reporting to the registry and the patients reported by a particular center. Moreover, the observational nature of the registry largely precludes interpretations of cause-effect relationships because of potential bias by indication.

## Summary

For more than a decade, the International Pediatric Peritoneal Dialysis Network (IPPN) has been collecting patient outcome data together with comprehensive clinical, biochemical, and treatment-related information from a large number of children undergoing CPD around the globe. These data have helped inform the management of a variety of clinical issues that are integral to the care of the pediatric PD population. It is hoped that the information that has been generated serves as a stimulus for further investigation and collaboration, all designed to improve the outcome of children who receive PD.
